# The Bile Acid Signaling Axis: Deciphering the Roles of FXR and TGR5 in Hepatic Steatosis, Fibrosis, and Cancer

**DOI:** 10.1002/prp2.70318

**Published:** 2026-09-29

**Authors:** Jie Liu, Kui Sun, Hongmei Liu, Meiyang Zhu, Chunmeng Li, Yuqi He, Lin Qin

**Affiliations:** ^1^ The Second Affiliated Hospital of Zunyi Medical University Zunyi China; ^2^ Guizhou Engineering Research Center of Industrial Key‐technology for Dendrobium Nobile, Guizhou Engineering Research Center for Orchid Medicinal Plant Breeding and Efficient Application Zunyi Medical University Zunyi Guizhou China

**Keywords:** bile acids, FXR, liver disease, TGR5

## Abstract

Bile acids (BAs) serve not only as emulsifiers for lipid digestion but also as essential signaling molecules, governing various physiological and pathological processes through their interaction with the farnesoid X receptor (FXR) and the takeda G protein‐coupled receptor 5 (TGR5). In recent years, the critical roles of FXR and TGR5 in ameliorating hepatic steatosis, modulating inflammatory responses, and regulating glucose and lipid metabolism, as well as in cholestasis, have gained significant recognition, positioning them as pivotal targets in liver disease research. This article offers a comprehensive review of the structural characteristics and physiological functions of both FXR and TGR5, highlighting their integral contributions to the pathogenesis and progression of various liver diseases, including metabolic dysfunction‐associated steatotic liver disease (MASLD), cholestatic liver disease (CLD), liver fibrosis, and liver cancer. Additionally, it examines the latest advancements in the research and development of pharmacological agents targeting FXR and TGR5, encompassing both agonists and antagonists, and explores their potential clinical applications. A thorough understanding of the FXR and TGR5 signaling pathways will provide a critical theoretical foundation for the development of treatment strategies for liver diseases.

**Abbreviations**
BAs: bile acids
BDL: bile duct ligation
BSEP: bile salt export protein
CA: cholic acid
CASP8: caspase 8
CDCA: chenodeoxycholic acid
CES1: carboxylesterase 1
Ch: cholesterol
CKMT2: mitochondrial creatine kinase 2
CLD: cholestatic liver disease
CPT‐1: carnitine palmitoyl transferase 1
CYP7A1: cholesterol 7‐αhydroxylase
CYP8B1: 12α‐hydroxylase
DCA: deoxycholic acid
DEN: diethylnitrosamine
FA: fatty acid
FGF15/19: fibroblast growth factor 15/19
FGFR4: fibroblast growth factor receptor 4
FOXA2: forkhead box protein A2
FXR: farnesoid X receptor
FXR: farnesol X receptor
GIP: glucose‐dependent insulinotropic polypeptide
GLP‐1: glucagon‐like peptide‐1
HCC: hepatocellular carcinoma
JNK: Jun N‐terminal kinase
LCA: lithocholic acid
LRH‐1: liver‐related homolog 1
LSECs: sinusoidal endothelial cells
MASH: metabolic dysfunction‐associated steatohepatitis
MASLD: metabolic dysfunction‐associated steatotic liver disease
MUFA: mono‐polyunsaturated fatty acids
NF‐κB: nuclear factor kappa‐light‐chain‐enhancer of activated B cells
NLRP3: NOD‐like receptor thermal protein domain associated protein 3
OCA: obeticolic acid
PBC: primary biliary cholangitis
PKA: protein kinase A
PKD: polycystic kidney disease
PLD: polycystic liver disease
PPARα: peroxisome proliferator‐activated receptor alpha
PSC: primary sclerosing cholangitis
PUFA: polyunsaturated fatty acids
SHP: hepatic small heterodimer partner
SREBP‐1c: sterol regulatory element‐binding protein 1c
STAT3: signal transducer and activator of transcription 3
TG: triglycerides
TGR5: takeda G protein‐coupled receptor 5
UCP1: uncoupling protein‐1

## 1 Introduction

FXR is a nuclear receptor predominantly expressed in the liver, intestine, and kidneys, and can be activated by BAs [1, 2]. The endogenous ligands of FXR include both conjugated and unconjugated BAs, and the ligand‐binding affinity varies depending on the BA type. The binding potency ranks as follows: chenodeoxycholic acid (CDCA) > deoxycholic acid (DCA) > lithocholic acid (LCA) > cholic acid (CA) [3, 4]. As the core sensor of BAs, FXR plays a crucial role in maintaining BA, cholesterol, carbohydrate, and lipid homeostasis, while also suppressing inflammatory responses and fibrotic processes through the regulation of downstream target genes [5, 6]. Consequently, FXR has emerged as an important therapeutic target for a variety of liver diseases—such as primary biliary cholangitis (PBC), liver fibrosis, metabolic dysfunction‐associated steatohepatitis (MASH), MASLD, as well as for metabolic disorders including hyperlipidemia and diabetes [7, 8]. TGR5 (also known as GPBAR1) is a membrane‐bound G protein–coupled receptor whose activation stimulates intracellular cyclic AMP (cAMP) production [9]. TGR5 is widely distributed in the liver, gallbladder, intestines, kidneys, spleen, brain, skeletal muscle, and brown adipose tissue [10]. Within the liver, TGR5 is mainly expressed in Kupffer cells, liver sinusoidal endothelial cells (LSECs), bile duct epithelial cells, natural killer T cells, and hepatocytes. In the intestine, TGR5 is primarily expressed by enteroendocrine cells in the colon, as well as by goblet and Paneth cells in the small intestine [11]. Its endogenous ligands are mainly BAs, and TGR5 is localized primarily on the plasma membrane [12]. The potency of BA‐induced TGR5 activation is ranked as follows: LCA>DCA>CDCA>CA [13]. Increasing evidence indicates that TGR5 activation plays a pivotal role in regulating glucose and lipid metabolism, immune and inflammatory responses, and communication along the gut‐liver axis [14, 15]. Figure 1 illustrates the regulatory roles of BA receptors FXR and TGR5 in liver diseases.

**FIGURE 1:**
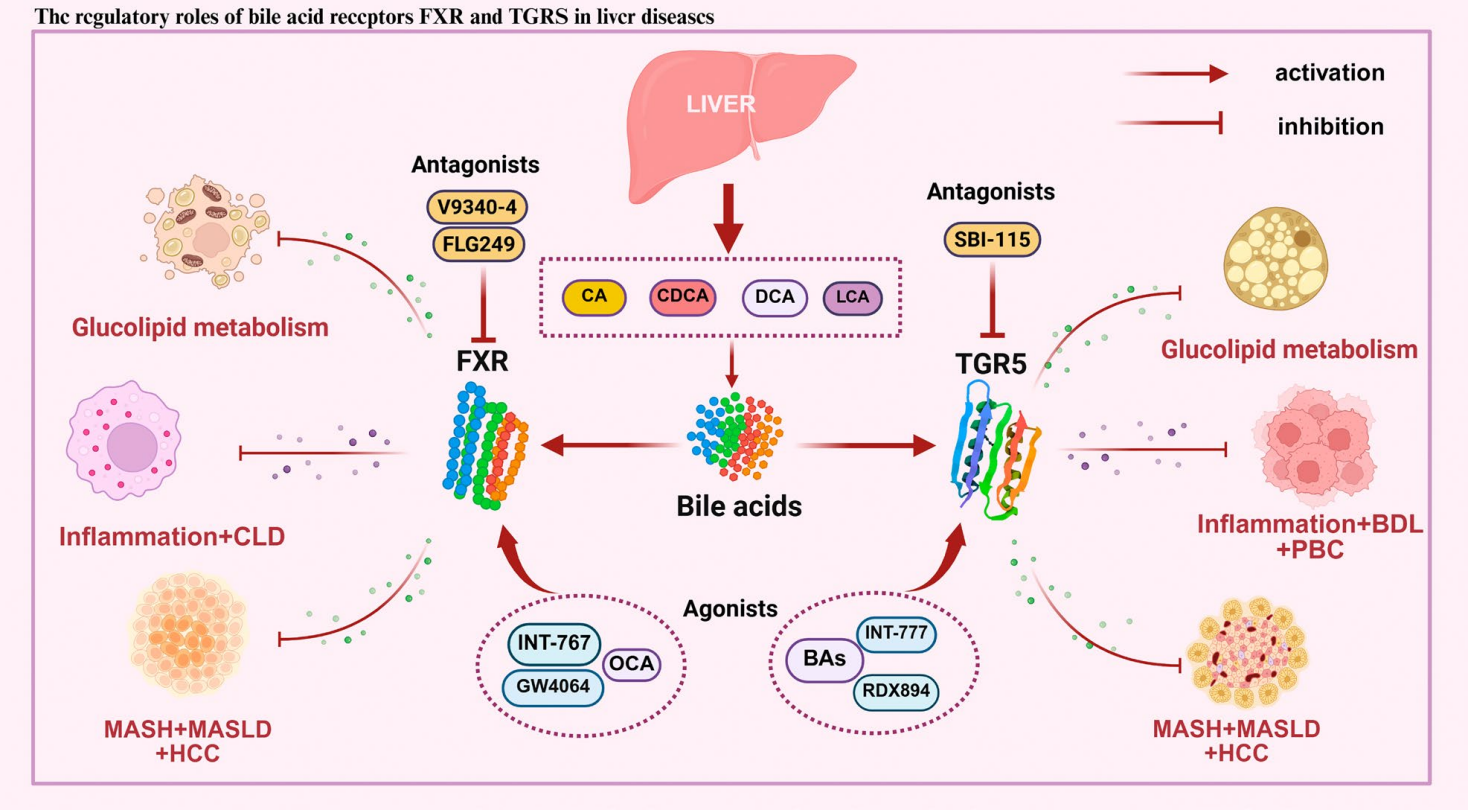
Schematic diagram of the regulatory roles of BA receptors FXR and TGR5 in liver diseases. BDL, bile duct ligation; CA, cholic acid; CDCA, chenodeoxycholic acid; CLD, cholestatic liver disease; DCA, deoxycholic acid; FXR, Farnesoid X receptor; HCC, hepatocellular carcinoma; LCA, lithocholic acid; MASH, metabolic dysfunction‐associated steatohepatitis; MASLD, metabolic dysfunction‐associated steatotic liver disease; PBC, primary biliary cholangitis; TGR5, Takeda G protein‐coupled receptor 5.

## 2 The Physiological Function of FXR and Its Research in Liver Diseases

### 2.1 The Physiological Functions of FXR

The activation of FXR achieves the following core physiological functions through a complex and precise transcriptional regulatory network:

#### 2.1.1 Regulating BA Homeostasis

BAs are primarily categorized into primary and secondary BAs. Primary/secondary BAs can conjugate with amino acids (such as glycine or taurine) to form conjugated BAs. The synthesis pathways for BAs mainly comprise the classical pathway and the alternative pathway [16]. The classical pathway that accounts for 75% of BAs synthesis is catalyzed by cholesterol 7‐α hydroxylase (CYP7A1), followed by steroid nucleus conversion, oxidative cleavage of its side chains, and regulation of BA synthesis and secretion by sterol 12α‐hydroxylase (CYP8B1) [16]. After synthesis in the liver, BAs enter the intestines. Under the influence of various gut microbiota and enzymes, most BAs are reabsorbed and returned to the liver [17].

The main way FXR regulates BAs is through hepatic small heterodimer partner (SHP), fibroblast growth factor 15/19 (FGF15/19), and Jun N‐terminal kinase (JNK). Activation of liver FXR receptors increases the expression of SHP [18], leading to inhibition of CYP7A1 and CYP8B1 [19], thereby reducing the synthesis and secretion of BAs. Meanwhile, the activation of intestinal FXR can act on fibroblast growth factor receptor 4 (FGFR4) through the FXR‐FGF15/FGF19 pathway [20], and then on JNK1/2, inhibiting the expression of CYP7A1 to reduce BA synthesis. In addition, FXR can stimulate bile secretion and BAs reabsorption by directly binding to the response elements of BAs secretion carriers or by regulating the expression of intestinal BAs binding proteins [21]. Notably, FXR exerts its effects on BAs most potently in the liver, followed by the small intestine and kidneys [22].

#### 2.1.2 Regulating the Balance of Glucose and Lipid Metabolism

*Inhibiting fat production*: FXR activation suppresses sterol regulatory element‐binding protein 1c (SREBP‐1c) expression via liver‐related homolog 1 (LRH‐1)‐mediated, SHP‐dependent signaling. SREBP‐1c is a master regulator of fatty acid (FA) and triglyceride (TG) synthesis enzymes (e.g., FA synthase), and its inhibition effectively reduces hepatic steatosis [23]. *Promoting FA oxidation*: FXR activation enhances peroxisome proliferator‐activated receptor alpha (PPARα) activity, upregulating its target gene carnitine palmitoyl transferase 1 (CPT‐1) and promoting FA *β*‐oxidation [24]. *Regulating the FA profile*: FXR activation lowers hepatic mono‐ polyunsaturated fatty acids (MUFA) levels by suppressing adipogenic genes (e.g., Dgat2, Lpin1, Scd1) independent of SHP‐SREBP1c, while reducing polyunsaturated fatty acids (PUFA) levels mainly via inhibition of lipid absorption [25]. *Regulating sugar metabolism*: FXR influences blood glucose through multiple mechanisms. Protein kinase A (PKA)‐mediated FXR phosphorylation binds to cAMP response element‐binding protein and activates gluconeogenic gene expression to lower glucose; however, glucagon‐activated forkhead box protein A2 (FOXA2) antagonizes this effect. Additionally, intestinal FXR enhances glucagon‐like peptide‐1 (GLP‐1) secretion, promoting insulin release and improving glucose tolerance [26, 27].

#### 2.1.3 Anti‐Inflammatory and Antioxidant Effects

FXR exerts anti‐inflammatory and antioxidant effects by suppressing NF‐κB (reducing IL‐6/IL‐1*β*) and activating NRF2‐mediated redox balance. Wedelolactone acts via the FXR‐BAs‐NF‐κB/NRF2 axis to reduce BA accumulation and inflammation [28], while melatonin upregulates FXR and inhibits TLR4/NF‐κB to alleviate Aflatoxin B1 induced liver injury [29]. NLRP3 inflammasome, upon activation, promotes caspase‐1 cleavage and subsequent maturation of IL‐1*β* and IL‐18, triggering inflammation and pyroptosis. FXR directly inhibits NLRP3‐ASC interaction and caspase‐1 activity via SHP, thereby alleviating hepatic inflammation [30]. In the intestine, BAs are transported via Bile salt export protein (BSEP) through the FXR‐SHP pathway, activating intestinal FXR, which in turn regulates BA synthesis and metabolism (CYP7A1, CYP8B1) through the FGF19‐FGF4‐JNK axis, ultimately improving inflammatory responses [31]. Figure 2 illustrates the physiological mechanism of the BA receptor FXR.

**FIGURE 2:**
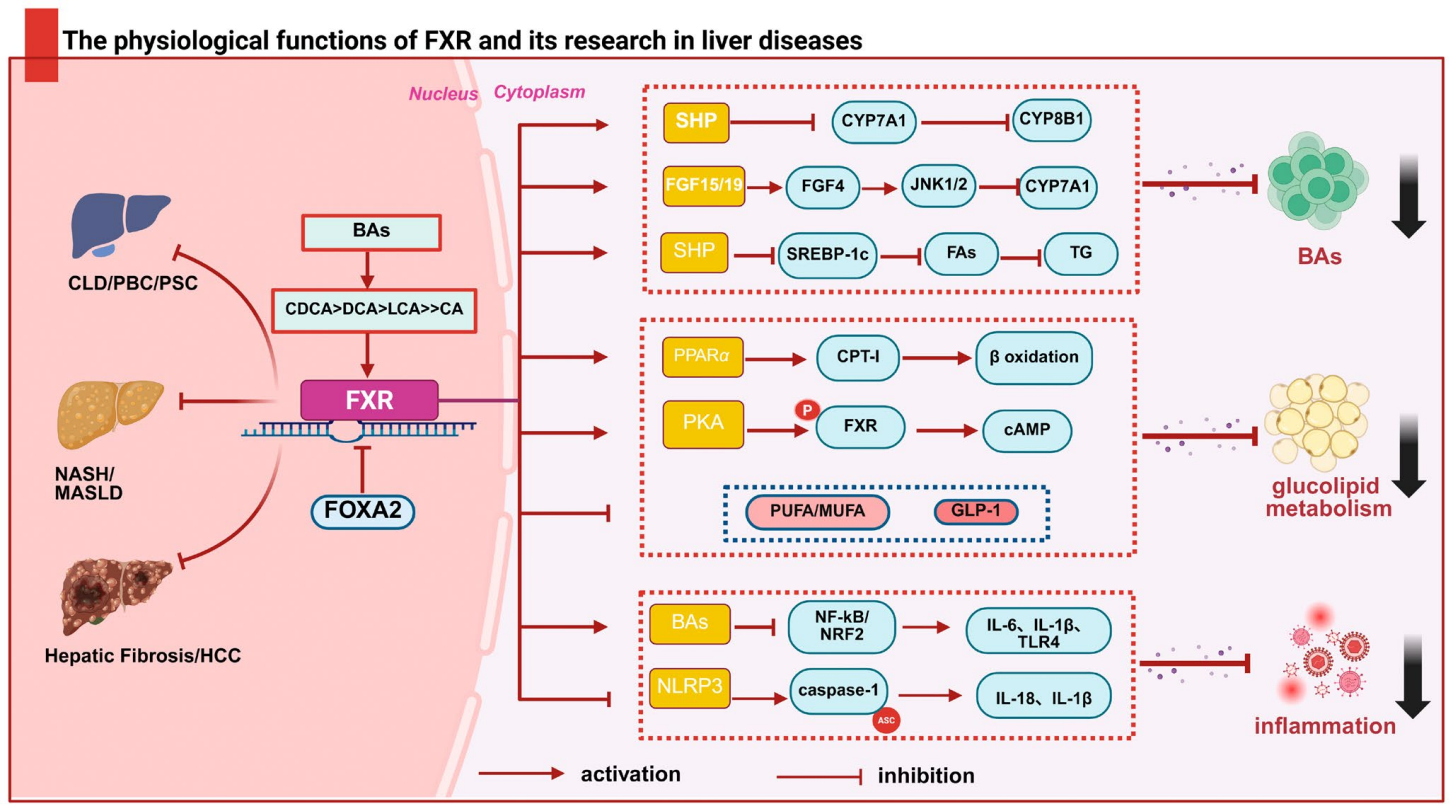
Schematic diagram of the physiological function mechanism of the BA receptor FXR. BAs, bile acids; CA, cholic acid; CDCA, chenodeoxycholic acid; CLD, cholestatic liver disease; CPT‐1, carnitine palmitoyl transferase 1; CYP7A1, cholesterol 7‐αhydroxylase; CYP8B1, 12α‐hydroxylase; DCA, deoxycholic acid; FAs, fatty acids; FGF15/19, fibroblast growth factor 15/19; FGFR4, fibroblast growth factor receptor 4; FOXA2, forkhead box protein A2; FXR, farnesoid X receptor; GLP‐1, glucagon‐like peptide‐1; HCC, hepatocellular carcinoma; JNK, Jun N‐terminal kinase; LCA, lithocholic acid; MASH, metabolic dysfunction‐associated steatohepatitis; MASLD, metabolic dysfunction‐associated steatotic liver disease; MUFA, mono‐ polyunsaturated fatty acids; NF‐κB, nuclear factor kappa‐light‐chain‐enhancer of activated B cells; NLRP3, NOD‐like receptor thermal protein domain associated protein 3; NRF2, nuclear factor erythroid 2‐related factor 2; PBC, primary biliary cholangitis; PKA, protein kinase A; PPARα, peroxisome proliferator‐activated receptor alpha; PSC, primary sclerosing cholangitis; PUFA, polyunsaturated fatty acids; SHP, hepatic small heterodimer partner; SREBP‐1c, sterol regulatory element‐binding protein 1c.

### 2.2 Research on the Role of FXR in Liver Diseases

#### 2.2.1 CLD

CLD is an inflammatory liver disease caused by the accumulation and blockage of large amounts of BAs. The common types mainly include primary sclerosing cholangitis (PSC) and PBC [32]. The main characteristics of PSC are narrowing of bile ducts, bile stasis, and fibrosis [33], as well as elevated liver enzyme indicators, abnormal BA metabolism, and changes in gut microbiota [34]. PSC patients lack effective drug treatment, and oral vancomycin has recently been identified as a potential treatment method. Long term vancomycin treatment can exacerbate bile stasis and liver fibrosis [35]. Activating intestinal FXR‐FGF15/19 signaling can improve vancomycin induced BA stasis and fibrosis, thereby effectively treating PSC [36]. Cilofexor is a non‐steroidal FXR agonist, and research has reported that Cilofexor can also improve PSC by activating FXR [37]. The main characteristic of PBC is the damage and destruction of bile ducts by biliary epithelial cells, leading to bile stasis [38]. FXR has long been a target for PBC treatment, and the FXR agonist obeticolic acid (OCA) has been clinically validated in multiple Phase II and III studies [39]. And another FXR agonist, Tropiferxor, enhances the adaptability of the FXR ligand binding region. In rodents, Tropiferxor effectively regulates FXR target genes in the liver and intestine, showing effective FXR target effects by transiently and dose‐dependent increasing FGF19, thereby improving the treatment of PBC [40].

#### 2.2.2 Liver Fibrosis and Liver Cancer

The primary pathological features of MASH are hepatocyte apoptosis and activation of hepatic stellate cells (HSCs), leading to liver fibrosis [41]. Current research has shown that FXR is a reliable target for intervention in the treatment of MASH disease [42]. Literature reports that FXR can bind to the caspase 8 (CASP8) through non‐canonical functions, thereby preventing the assembly of death receptor complexes, inhibiting CASP8 cleavage, and suppressing hepatocyte apoptosis, thereby exerting anti‐liver fibrosis and avoiding HSC activation [43]. In addition, carboxylesterase 1 (CES1) is also a key regulatory factor in the pathogenesis of MASH. CES1 is highly expressed in hepatocytes and is the main enzyme for lipid hydrolysis. FXR regulates the expression level of CES1 through FXRE, and CES1 has the function of partially mediating FXR to inhibit lipid deposition and protect hepatocytes, which can inhibit the development of MASH fibrosis [44].

Hepatocellular carcinoma (HCC) is a common form of liver cancer [45]. In liver tumors from FXR^−/−^ mice, the expression of inflammatory factors, cell cycle markers, and the myeloma oncogene is increased [46]. The accumulation of BAs and the increased expression of *β*‐catenin target genes Myc and Ccnd1 mRNAs were mainly observed in FXR^−/−^ HCC mice [47]. FXR activation also reduces HCC transcriptional activity by binding to *β*‐catenin and disrupting the core *β*‐catenin‐TCF4 complex [48]. Research has also demonstrated that FXR can regulate BAs through the FGF15/19‐FGFR4‐*β*‐klotho axis to inhibit HCC progression [49]. Besides, activation of FXR can also suppress HCC via the JAK2‐STAT3 pathway [50].

#### 2.2.3 Masld

MASLD arises from multiple concurrent pathogenic factors, including lipid metabolism disorders and aberrant secretion of cytokines and adipokines [51]. It is characterized by excessive hepatic triglyceride (TG) accumulation, disrupted fat and FA uptake, and impaired oxidation of very low‐density lipoprotein entering the systemic circulation [52]. Activating FXR is an effective strategy to improve MASLD/MASH through the following mechanisms:

1. *Regulation of lipid metabolism*. FXR inhibits fat production via the FXR‐SHP‐SREBP1c pathway, promotes FA oxidation via the FXR‐PPARα pathway [23, 24], and induces expression of CYP3A4/A11, Sulfotransferase 2A1, and UDP‐glucuronosyltransferase 2B4 to enhance BA detoxification and excretion [53].
2. *Reciprocal regulation with SIRT1*. SIRT1, a histone deacetylase, inhibits FA synthesis and oxidation; its deficiency contributes to MASLD [54]. FXR increases SIRT1 expression by inducing SHP, which suppresses microRNA‐34a‐mediated negative regulation of SIRT1. In turn, SIRT1 improves MASLD by promoting FXR deacetylation, enhancing its stability and nuclear translocation, thereby forming a positive regulatory loop [54, 55, 56].
3. *Maintenance of intestinal barrier function*. Activation of intestinal FXR is critical for MASLD management [57]. It maintains intestinal epithelial tight junctions by inhibiting IFNγ expression [58], preserves gut vascular barrier integrity via the Wnt/*β*‐catenin pathway [59], and upregulates iNOS, IL‐18, and Ang1 to enhance intestinal antibacterial defense and repair [60].

### 2.3 Research on FXR Agonists and Antagonists

The activation of FXR has been proven to have a protective effect on various liver diseases, so the development and utilization of FXR agonists have been widely promoted. At present, a considerable number of FXR agonists have been applied in clinical practice, mainly divided into endogenous, synthetic, and natural categories. BAs are endogenous FXR agonists, and primary BAs mainly include CA, CDCA, etc. Secondary BAs include DCA, LCA, and GUDCA [61]. CDCA activation of intestinal FXR can improve glucose metabolism associated with polycystic ovary syndrome [62], DCA could induce intestinal inflammation [63], LCA is a FXR antagonist with partial agonist activity that can induce cholestatic itching or reduce the expression of BSEP under different physiological conditions [64].

Currently, numerous synthetic FXR agonists are available, such as OCA, GW4064, and INT‐767. OCA is a CDCA derivative and the first FXR agonist approved for PBC. Additionally, it has demonstrated potential for improving liver histology and biochemistry in MASH treatment [65]. However, given persistent concerns about safety and lack of efficacy in the COBALT trial, OCA was voluntarily withdrawn by the manufacturer in September 2025 and is no longer available for prescription for PBC treatment in the United States [66]. GW4064 is a potent, highly selective agonist that significantly reduces lipid levels in MASLD patients [67]. INT‐767 is a dual FXR/TGR5 agonist capable of improving histological features of MASLD/MASH and renal fibrosis [68]. Other synthetic FXR agonists include Cilofexor, Tropiferxor, Vonafexor, and other novel synthetic agonists that are currently in different stages of clinical development with the aim of optimizing efficacy, selectivity, and safety [37, 40, 69]. Natural product agonists: Various natural compounds (such as glycosides, salidroside, flavonoids, polyphenols, etc.) have been proven to have FXR agonist activity and are potential sources of drugs [70].

FXR antagonists have been studied less extensively and are currently limited in variety. Endogenous antagonists include T‐*β*MCA, TUDCA, and GUDCA, while synthetic options encompass FLG249 and V023‐9340 [71, 72]. These antagonists may hold therapeutic value under specific pathological conditions, such as certain types of cholestasis or scenarios requiring temporary blockade of FXR signaling, and represent a significant avenue for future research. Table 1 lists the details of FXR agonists and antagonists.

**TABLE 1:** List of commonly used agonists and antagonists of FXR.

Drug name	Receptor selectivity	Clinical indication	Stage of development	Major adverse effects	Principal limitations	Description
OCA	Highly selective steroidal FXR agonists	PBC (previously approved); MASH [73]	Approved for delisting (approved in 2016 and delisted in 2025 in the United States)	Hepatotoxicity; Severe pruritus; Elevated LDL/low HDL [74]	The risk of liver toxicity far outweighs the benefits	Tmax of 1.5 h, half‐life of 24 h, and 87% fecal excretion. The recommended dosage is 5–10 mg. The high dosage (25/50 mg) increases the toxicity [74] Reduce ALT and alleviate liver fibrosis OCA can lead to liver failure and death risk, FDA requires delisting
GW4064	Non‐steroidal FXR agonists capable of activating both mice and human FXR	It reduces serum triglycerides and can be used for the treatment of MASLD [75]	Preclinical study	Weight gain, fatty liver and insulin resistance (i.e., leading to obesity and diabetes) [76]	Oral bioavailability is extremely low. Preclinical research tool drugs [77]	The oral bioavailability is only 10%, half‐life of 3.5 h GW4064 is a classic tool drug specifically designed for scientific research and has never entered clinical trials. Widely used as a “standard” or positive control [77]
INT‐767	FXR/TGR5 dual agonist	MASH, anti‐HBV [78]	Terminated at the clinical Phase I stage	N/A	N/A	Highly stable for enzyme modifications of Phase I and Phase II. Intravenous injection is possible [79]
Cilofexor	Highly selective nonsteroidal FXR agonists	Developed for MASH and PSC [80]	Termination of Phase III	Itching, elevated liver enzymes, headache, fatigue [32]	Termination of invalidity of PSC in mid‐term of Phase III [1]	Oral absorption, half‐life of 2‐13 h, moderate fat meal reduces AUC by 21%–45%. Patients with liver function impairment need to adjust the dosage [81]
Tropiferxor	Highly selective FXR agonist	Mainly used for MASLD/MASH [80]	Phase II clinical trial has been completed, and the research has been terminated	Itching, headache, nausea	Not meeting the Phase III start‐up criteria. Failed to demonstrate clinically significant efficacy at the histological endpoint of MASH (MASH remission or fibrosis improvement) [82]	Oral absorption, peak time is about 4 h, half‐life is 13.5–21.9 h. Mainly cleared through oxidative metabolism, with the prototype drug as the main circulating component (> 92%). Fecal excretion is the main pathway (about 65%). Food can increase exposure by about 60% [83]
Vonafexor	Non‐steroidal FXR agonists with renal targeting properties	MASH; Alport syndrome (scheduled to start Phase III in 2026); CKD combined with MASH (Phase II ongoing) [84, 85]	During Phase II clinical trials, nine clinical studies have been completed	Itching and elevated LDL cholesterol [69]	Not yet terminated, research and development is ongoing	Oral administration, once daily. Preclinical: Reversing renal fibrosis, delaying CKD progression, and prolonging the lifespan of Alport syndrome mice. Phase II clinical trial: reversing the annual decline rate of eGFR in patients with Alport syndrome
FLG249	Non‐steroidal FXR antagonists	Significantly reduce liver triglycerides (TG) and serum total cholesterol (Ch) [86]	Preclinical study	N/A	It is mainly used as a research tool‐drug to study the biological functions of FXR and to explore new therapies for metabolic diseases	In animal experiments, it is administered orally at a dose of 10 mg/kg twice daily. FLG249 has a tendency towards ileal distribution and tends to accumulate in the intestine [72]
V023‐9340	Intestinal FXR selective antagonist	For potential treatment of MASLD, NASH [71]	Preclinical study	N/A	All efficacy and safety data are limited to animal models, with human data completely lacking	Prioritizing distribution in intestinal tissue helps achieve targeted intestinal effects

## 3 The Physiological Function of TGR5 and Its Research in Liver Diseases

### 3.1 Physiological Functions of TGR5

#### 3.1.1 Regulating Sugar and Lipid Metabolism and Energy Metabolism

TGR5 is a key mediator of metabolic regulation. Its activation promotes GLP‐1 secretion from intestinal endocrine cells, stimulating insulin release, enhancing insulin sensitivity, and maintaining glucose homeostasis [87, 88, 89]. TGR5 activation also improves glucose tolerance and reverses high‐fat diet‐induced hyperglycemia [90, 91], and may modulate glucose metabolism via polypeptide (GIP) regulation [92]. Centrally, TGR5 in hypothalamic steroidogenic factor‐1 neurons enhances insulin signaling and systemic insulin sensitivity [93]. In the heart, TGR5 limits FA uptake by regulating DHHC4, thereby preventing lipid accumulation in diabetic cardiomyopathy [94]. In lipid metabolism and energy balance, TGR5 activation induces white adipose tissue browning, increasing thermogenesis and energy expenditure [95, 96]. This involves two mechanisms: (1) enhancing thyroid hormone activity (T4‐to‐T3 conversion) via cAMP‐PKA signaling, which upregulates UCP1 expression and promotes mitochondrial thermogenesis [97, 98]; and (2) inducing Mitochondrial creatine kinase 2 (CKMT2) expression to activate the futile creatine cycle, establishing a UCP1‐independent thermogenic pathway [99, 100]. Collectively, these actions alleviate obesity and related metabolic disorders.

#### 3.1.2 Mediate Anti‐Inflammatory and Immune Regulation

The activation of TGR5 exerts significant anti‐inflammatory and immune regulatory effects through multiple mechanisms. Research has shown that secondary BA DCA can inhibit NF‐κB and NLRP3 inflammasome activation through the TGR5‐cAMP‐PKA axis, thereby alleviating *Staphylococcus aureus* induced mastitis [101]. Similarly, natural bear bile powder can inhibit LPS induced neuroinflammation through the TGR5/AKT/NF‐κB pathway [102].

At the level of immune cells, TGR5 plays a crucial regulatory role in macrophage polarization. Its activation inhibits NLRP3 inflammasome‐mediated caspase‐1 activation, preventing macrophages from polarizing towards the pro‐inflammatory M1 phenotype and potentially promoting their transition to the anti‐inflammatory M2 phenotype, thereby improving inflammatory states [103]. In a sepsis model, traditional Chinese medicine Pien‐Tze‐Huang effectively inhibits the NF‐κB and MAPK pathways downstream of TLR4 by activating the TGR5/STAT3/A20 signaling axis, reducing the release of pro‐inflammatory factors such as TNF‐α, IL‐1*β*, and IL‐6 [104].

#### 3.1.3 Regulating BA Synthesis and Metabolism

As a membrane‐bound BA receptor, TGR5 rapidly responds to extracellular BA signals [105], yet can be shielded by the bicarbonate‐rich glycocalyx from BA overstimulation [106]. Activation of TGR5 enhances lipid metabolism and modulates BA homeostasis through the TGR5‐cAMP‐PKA‐D2/CKMT2 pathways, along with other mechanisms involved in BA synthesis and metabolism [98, 100]. Functionally, TGR5 couples BA uptake with chloride secretion: activation increases intracellular cAMP and stimulates cystic fibrosis transmembrane conductance regulator‐mediated Cl^−^ efflux, driving bicarbonate‐rich fluid secretion and affecting BA excretion [107]. This modulation of biliary Cl^−^/HCO_3_^−^ transport attenuates hepatobiliary toxicity and facilitates BA enterohepatic recirculation [108]. In TGR5^−/−^ mice, the BA pool is reduced and bile flow diminished [109]. TGR5 also downregulates CYP8B1 expression, decreasing the 12α‐hydroxylated/non‐12α‐hydroxylated BA ratio and reducing BA pool hydrophobicity [110]. Consistently, TGR5^−/−^ mice show elevated CYP8B1 and hydrophobic 12α‐hydroxy BAs, whereas WT mice exhibit significantly lower levels [111]. Beyond BA metabolism, TGR5 engagement modulates NF‐κB, NLRP3, GLP‐1, and white adipose tissue pathways, collectively improving glucose/lipid homeostasis and attenuating inflammation. Figure 3 illustrates the physiological mechanisms of TGR5 signaling.

**FIGURE 3:**
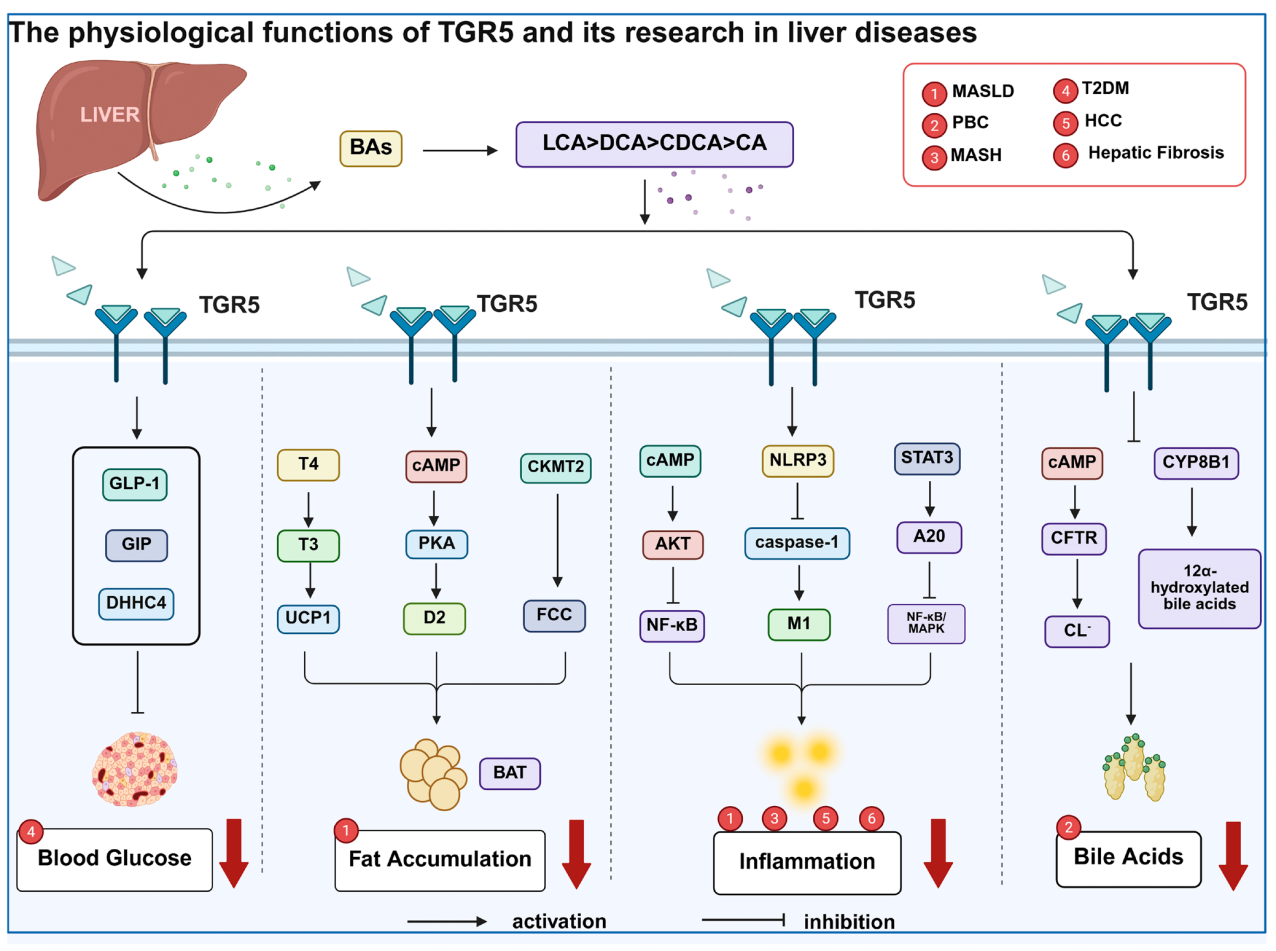
Physiological functional mechanism diagram of BA receptor TGR5. BAs, bile acids; CA, cholic acid; CDCA, chenodeoxycholic acid; CYP8B1, 12α‐hydroxylase; DCA, deoxycholic acid; GIP, glucose‐dependent insulinotropic polypeptide; GLP‐1, glucagon‐like peptide‐1; HCC, hepatocellular carcinoma; LCA, lithocholic acid; MAPK, mitogen‐activated protein kinase; MASH, metabolic dysfunction‐associated steatohepatitis; MASLD, metabolic dysfunction‐associated steatotic liver disease; NF‐κB, nuclear factor kappa‐light‐chain‐enhancer of activated B cells; NLRP3, NOD‐like receptor thermal protein domain associated protein 3; PBC, primary biliary cholangitis; PKA, protein kinase A; STAT3, signal transducer and activator of transcription 3; T2DM, Type 2 diabetes mellitus; TGR5, takeda G protein‐coupled receptor 5; UCP1, uncoupling protein‐1.

### 3.2 Research on the Role of TGR5 in Liver Diseases

#### 3.2.1 Masld

TGR5 has emerged as a potential therapeutic target for MASLD and associated metabolic disorders [112]. Its mechanisms for improving MASLD are multifaceted: First, it enhances insulin resistance by stimulating GLP‐1 secretion [113]; Second, it activates eNOS via the cAMP pathway, increasing the production of protective nitric oxide (NO) [114]; Third, it reduces hepatic inflammation by inhibiting macrophage M1 polarization through PKA‐mediated NLRP3 inflammasome ubiquitination, or by suppressing macrophage migration via the mTORC1 signaling pathway [115, 116].

#### 3.2.2 CLD

The expression of TGR5 in biliary epithelial cells is crucial for their secretion, proliferation, and tight junction integrity. However, its function is complex, and inhibiting TGR5 may delay the progression of PSC in some cases [117]. In the bile duct ligation (BDL) induced cholestasis model, TGR5 gene knockout exacerbated oxidative stress and M1 polarization in Kupffer cells, while TGR5 agonists reduced oxidative damage and inflammation by inhibiting NF‐κB and activating the Nrf2/HO‐1 pathway, exerting a protective effect [118]. Similarly, activating TGR5 may also alleviate PBC by protecting bile duct cells [119].

#### 3.2.3 Liver Fibrosis and Liver Cancer

In liver fibrosis, the TGR5 agonist LCA can improve fibrosis by inhibiting TGF‐*β* signaling and glycolysis, promoting macrophage differentiation towards the M2 phenotype, and regulating natural killer T cells' activity [120]. Another agonist, BAR501, improved liver fibrosis in a TGR5‐dependent manner while reversing liver injury and vascular inflammation in MASH mouse models [121]. Additionally, activation of TGR5 in liver LSECs promotes endothelial nitric oxide synthase (eNOS) phosphorylation, increasing the production of vasodilatory factors such as NO and hydrogen sulfide. This may help improve hepatic microcirculation and suppress fibrosis [122]. TGR5 activation also inhibits HSC activation by regulating mitochondrial dynamics [123].

TGR5 exhibits anti‐cancer properties in HCC. TGR5 deficient mice are more sensitive to the induction of liver cancer by the chemical carcinogen diethylnitrosamine (DEN), and their inflammatory response is enhanced. Also TGR5 activation can inhibit tumor development by suppressing the STAT3 pathway [124]. Furthermore, inhibiting the α7‐nicotinic acetylcholine receptor (α7‐nAChR)/TGR5/JAK2/STAT3 signaling pathway has also been shown to slow down the progression of HCC [125].

### 3.3 Research Progress on TGR5 Agonists and Antagonists

Currently, TGR5 agonists encompass three major categories: BAs analogues, naturally occurring compounds, and synthetic molecules. As the earliest identified class of TGR5 ligands, BAs exhibit structure‐dependent agonistic activity. Hydrophobic species, particularly LCA, act as potent agonists, and their tauroconjugation further potentiates their efficacy; however, poor target selectivity and inherent cytotoxicity have precluded their direct clinical translation [126]. In parallel, several naturally occurring pentacyclic triterpenoids, including betulinic acid, oleanolic acid, and ursolic acid—have been characterized as TGR5 agonists [127]. Among synthetic agonists, INT‐777 is the most extensively studied, having demonstrated efficacy in ameliorating hepatic steatosis and insulin resistance in obese murine models [128]. Notably, RDX894, a next‐generation synthetic agonist, exhibits a favorable safety profile with no reported gallbladder emptying inhibition or hepatopancreatic toxicity, while retaining potent activity against insulin resistance and hepatic steatosis, positioning it as a promising therapeutic candidate for MASH/MASLD [129].

Research on TGR5 antagonists remains in its infancy. SBI‐115, the first reported antagonist, has been shown to exacerbate intestinal inflammation by inhibiting the TGR5/*β*‐arrestin2 pathway and inducing mitochondrial structural abnormalities in pancreatic cancer cells [130, 131]. SBI‐364 is a novel competitive TGR5 receptor antagonist. Studies have shown that SBI‐364 slows down the formation of hepatic and renal cysts by targeting TGR5 in the liver and kidneys. It can be considered as a novel treatment option for polycystic kidney disease (PKD) and related polycystic liver disease (PLD) [132]. Furthermore, TGR5 antagonists have demonstrated potential applications in exploratory studies, such as inhibiting liver cyst formation and alleviating neuropathic pain. However, their comprehensive biological effects and therapeutic value require further investigation. Table 2 lists the details of TGR5 agonists and antagonists.

**TABLE 2:** List of commonly used agonists and antagonists of TGR5.

Drug name	Receptor selectivity	Clinical indication	Stage of development	Major adverse effects	Principal limitations	Description
INT‐777	Semi synthetic secondary BA analog, TGR5 selective agonist	Preclinical studies mainly focused on T2DM, obesity, and MASLD [133]	Only used for preclinical basic research and has not yet entered the human clinical trial stage [128]	N/A	Lack of clinical data, only used as a preclinical tool drug	The plasma protein binding rate is about 62%; Highly stable for enzymatic hydrolysis, mainly discharged into bile in its original form. It stimulates GLP‐1 and insulin secretion, suppresses inflammatory response, and decreases triglyceride accumulation in hepatocytes [133]
RDX894	TGR5 potent agonist	It has therapeutic potential for MASLD/MASH patients	Preclinical study	It can inhibit the emptying of the gallbladder (which may increase the risk of gallstones) and affect heart rate and blood pressure [113]	Lack of human data	In mice fed with a Western diet (inducing MASLD model), RDX8940 effectively induces intestinal L cells to secrete intestinal proinsulin (including GLP‐1, GLP‐2, and PYY), thereby improving liver steatosis and insulin sensitivity [113]
SBI‐115[130131]	Highly selective TGR5 antagonist	Polycystic liver disease (PLD)	Preclinical study	N/A	Only used as a tool drug	SBI‐115 can be administered via intracerebral microinfusion or perineural injection of the sciatic nerve [134]
SBI‐364	TGR5 competitive antagonist	Polycystic liver disease and kidney disease and cholangiocarcinoma [132]	Preclinical study	N/A	Lack of human body data	Its development aims to simultaneously treat PKD and its associated PLD [132]

## 4 Interaction Between FXR and TGR5

FXR and TGR5 are two central receptors mediating BA signal sensing, which assemble a sophisticated multi‐layer regulatory network to jointly sustain metabolic homeostasis [135].

First, the two receptors coordinately maintain BA homeostasis. As its master regulator, FXR inhibits BA synthesis via negative feedback: hepatic FXR activation upregulates SHP to repress CYP7A1 transcription, whereas intestinal FXR induces FGF15/19. Secreted FGF15/19 travels via the portal vein to the liver, binds the FGFR4/β‐Klotho complex, and suppresses CYP7A1 to reduce BA synthesis [136]. By contrast, TGR5 mainly mediates BA‐dependent intermediate metabolism, exerting complementary functions in energy expenditure and glucose homeostasis [137].

Second, FXR and TGR5 act as critical mediators for BA signal transduction within the gut‐liver axis [138]. In the intestine, BAs activate intracellular FXR in enterocytes and trigger secretion of FGF15/19 into the portal venous circulation. As an endocrine signal, FGF15/19 is delivered to the liver to modulate hepatic BA synthesis, lipid metabolism, and gluconeogenesis [136, 139]. The function of TGR5 within the enterohepatic axis predominantly resides in metabolic regulation. Upon activation by BAs, TGR5 located on intestinal L endocrine cells stimulates the secretion of GLP‐1, which subsequently promotes insulin secretion and alleviates insulin resistance [139]. In addition, intestinal microbiota remodel BA structures to generate microbial BA conjugates capable of activating both FXR and TGR5, further enriching the signaling regulatory network of the enterohepatic axis [140].

Finally, FXR and TGR5 exert synergistic and complementary effects in metabolic regulation. FXR modulates gene transcription primarily via nuclear receptor signaling pathways, whereas TGR5 rapidly regulates cellular function through G protein‐coupled receptor signaling cascades [139]. During liver regeneration, FXR facilitates hepatocyte proliferation and cell cycle progression; meanwhile, TGR5 modulates BA hydrophobicity and promotes urinary excretion of BAs, and the two receptors collectively ensure unimpeded liver regeneration [136]. Single‐gene knockout of either FXR or TGR5 results in delayed liver regeneration in mice, indicating that these two receptors possess partial functional compensation yet are not fully interchangeable [141]. In intestinal epithelial cells, FXR generally exerts antiproliferative effects by upregulating cell‐cycle inhibitors such as p21. In contrast, TGR5 promotes cell proliferation through crosstalk activation of the cAMP–PKA pathway and EGFR signaling. These two opposing effects constitute a delicate balance for the maintenance of intestinal epithelial homeostasis [142].

## 5 Conclusion and Prospect

This article systematically reviews the core roles of BA nuclear receptor FXR and membrane receptor TGR5 in liver physiology and disease progression. Studies have demonstrated that FXR holds considerable therapeutic potential for ameliorating various liver diseases, including cholestasis, MASLD, MASH, and HCC. Its mechanisms involve regulating BA homeostasis, inhibiting classic inflammatory pathways such as NF‐κB/NLRP3, and modulating glucose and lipid metabolism pathways like SREBP‐1c/PPAR‐α/GLP‐1. Complementarily, TGR5 plays a crucial role in regulating bile secretion, stimulating GLP‐1 release, inhibiting inflammatory responses mediated by NF‐κB/NLRP3/STAT3, and protecting bile duct cells through activation of signaling axes such as cAMP/PKA. Together, they form an important signaling network to resist liver damage.

Despite significant advances in FXR and TGR5 research, their clinical translation still faces several challenges. First, the variety of FXR‐ and TGR5‐targeted drugs (including agonists and antagonists) that have reached clinical application remains limited. Most agents under development suffer from issues such as low bioavailability, short duration of action, notable side effects, or poor clinical translation rates. Moreover, the development of dual agonists/antagonists capable of synergistically modulating both FXR and TGR5 signaling remains unexplored, which constrains their potential for comprehensive therapeutic intervention. Second, FXR and TGR5 are widely distributed throughout the body, and their precise physiological and pathological functions‐as well as their pharmacological effects‐in various tissues and organs (e.g., heart, kidney, brain, lungs) have not been fully elucidated. Current research remains largely liver‐centric, lacking a systematic perspective and thereby introducing potential uncertainties for systemic drug applications.

Meanwhile, FXR function has tissue, cellular, and pathological dependencies. There is a significant difference in the interaction between the liver and intestine, with only 11% of the mice liver intestine FXR sharing binding sites, indicating a completely different regulatory gene network [143]. Single organ knockout of FXR phenotype is mild, while double knockout can induce severe cholestatic liver injury, fibrosis, and abnormal proliferation, indicating that enterohepatic FXR synergistically maintains homeostasis and cannot fully compensate for each other. Activating FXR can improve metabolic and immune diseases, while inhibiting FXR in cerebral ischemia, pancreatic cancer, and other scenarios is more beneficial [144]. These factors complicate the development of FXR‐targeted therapies for clinical benefits. Pruritus, dyslipidemia, and hepatotoxicity represent the most frequent adverse reactions of FXR agonists, constituting major bottlenecks restricting their advancement in clinical trials. The development of numerous FXR agonists has been terminated due to unfavorable risk–benefit profiles, wherein adverse effects outweigh therapeutic efficacy. This poses challenges for the development of next‐generation FXR agonists while identifying novel directions for breakthrough research.

In addition, studies have revealed that TGR5 exerts dual effects on cholangiocyte proliferation and PSC. Under physiological conditions or cholestasis, TGR5 activation mediates protective adaptive responses. By contrast, this signaling pathway can be hijacked in specific pathological microenvironments to promote disease progression [119, 145]. TGR5 serves as a pivotal mediator of BA‐dependent cholangiocyte proliferation, although the exact mechanism underlying its pro‐proliferative effect remains controversial. In TGR5^−/−^ mice, cholangiocyte proliferation induced by BA treatment or BDL is markedly attenuated [146]. Nevertheless, sustained or excessive activation of TGR5 signaling becomes pathogenic, triggering aberrant cholangiocyte proliferation and cyst expansion in polycystic liver disease [145]. On the other hand, the expression pattern and function of TGR5 in PSC are also under debate, cholangiocyte‐specific downregulation of TGR5 is considered a major cause of impaired cytoprotection in PSC [117]. However, TGR5 is highly expressed in CD206^+^ macrophages isolated from PSC injured livers, indicating that TGR5 not only mediates local protective effects in cholangiocytes but may also regulate immune cell function [117]. Accordingly, it remains unclear whether TGR5 contributes to PSC progression via reduced protective capacity resulting from its cholangiocyte‐specific downregulation or through inducing global hepatic immune dysregulation. These questions warrant further in‐depth investigation into our subsequent studies.

Looking ahead, research in this field should focus on the following directions:

1. *Deepening mechanistic insight*: Further elucidating the fine‐tuned regulatory networks and crosstalk between FXR and TGR5 in hepatic and extrahepatic tissues and clarifying their cell‐type‐specific functions under particular pathological conditions.
2. *Promoting drug innovation*: Developing novel FXR and TGR5 modulators, including single and dual‐target agents—with improved efficacy, selectivity, and safety profiles, and leveraging advanced drug delivery systems to enhance tissue targeting and therapeutic effectiveness.
3. *Explore combination strategies*: Investigate combination regimens of FXR and TGR5 agonists with other targeted agents such as GLP‐1 receptor agonists, so as to enhance the target‐specific efficacy of FXR/TGR5 agonists via synergistic effects and alleviate adverse reactions induced by monotherapy.
4. *Strengthening clinical translation*: Conducting rigorously designed clinical trials to validate the safety and efficacy of existing and future drug candidates and facilitating the translation of foundational research into tangible clinical treatments.

## Author Contributions

**Jie Liu:** writing – original draft, writing – review and editing, funding acquisition, project administration. **Kui Sun:** software. **Hongmei Liu:** methodology, investigation. **Meiyang Zhu:** validation, formal analysis. **Chunmeng Li:** validation, investigation. **Yuqi He:** project administration, resources, writing – review and editing. **Lin Qin:** project administration, writing – review and editing, resources.

## Funding

The study was supported by the National Nature Science Foundation of China (U24A20793, 82260843), Science and Technology Research on Traditional Chinese Medicine and Ethnic Medicine in Guizhou Province (QZYY‐2025‐073), Zunyi City Science and Technology Planning Project (ZSKH HZ (2025) 131), the Department of Science and Technology of Guizhou Province (QKHPTRC‐CXTD [2023]024).

## Ethics Statement

The authors have nothing to report.

## Consent

The authors have nothing to report.

## Conflicts of Interest

The authors declare no conflicts of interest.

## Data Availability

The authors have nothing to report.
